# Syntactic Comprehension of Relative Clauses and Center Embedding Using Pseudowords

**DOI:** 10.3390/brainsci10040202

**Published:** 2020-03-31

**Authors:** Kyung-Hwan Cheon, Youngjoo Kim, Hee-Dong Yoon, Ki-Chun Nam, Sun-Young Lee, Hyeon-Ae Jeon

**Affiliations:** 1 Department of Brain and Cognitive Sciences, Daegu Gyeongbuk Institute of Science and Technology (DGIST), Daegu 42988, Korea; 2 Department of Korean Language, Kyung Hee University, Yongin 17104, Korea; 3 Department of Psychology, Korea University, Seoul 02841, Korea; 4 Department of English, Cyber Hankuk University of Foreign Studies, Seoul 02450, Korea; 5 Partner Group of the Max Planck Institute for Human Cognitive and Brain Sciences at the Department of Brain and Cognitive Sciences, DGIST, Daegu 42988, Korea

**Keywords:** syntactic hierarchy, merge, embedding, self-paced reading

## Abstract

Relative clause (RC) formation and center embedding (CE) are two primary syntactic operations fundamental for creating and understanding complex sentences. Ample evidence from previous cross-linguistic studies has revealed several similarities and differences between RC and CE. However, it is not easy to investigate the effect of pure syntactic constraints for RC and CE without the interference of semantic and pragmatic interactions. Here, we show how readers process CE and RC using a self-paced reading task in Korean. More interestingly, we adopted a novel self-paced pseudoword reading task to exploit syntactic operations of the RC and CE, eliminating the semantic and pragmatic interference in sentence comprehension. Our results showed that the main effects of RC and CE conform to previous studies. Furthermore, we found a facilitation effect of sentence comprehension when we combined an RC and CE in a complex sentence. Our study provides a valuable insight into how the purely syntactic processing of RC and CE assists comprehension of complex sentences.

## 1. Introduction

Constructing relative clauses (RC) is one of the core processes in complex sentence formation and has been intensely studied in the field of psycholinguistics [1,2,3,4]. The complexity and diversity of RC construction across different natural languages have provoked a plethora of cross-linguistic studies as well as psycholinguistic studies, focusing on a cognitive approach in the hope of building a universal explanation for the various RC structures of different languages. For example, an asymmetry of comprehension difficulties between subject-extracted RC and object-extracted RC [5,6,7,8,9,10,11,12,13,14,15,16,17,18,19,20,21] has been investigated in different languages. While no single account encompasses the varying nature of RC constraints, considerable evidence for experience-based approaches such as the surprisal theory has steadily accumulated [7,15,20,22,23,24,25,26,27,28,29,30,31].

Center embedding (CE), in addition to RC, presents another interesting aspect for understanding and creating complex sentences for parsers. A study of artificial grammar learning [32] proposed that a recursive structure by CE in natural languages is the symbolic computation unique to human language. CE is established by inserting a subordinate clause within a superordinate clause [33]. While expectation-based approaches on RC construction emphasize cognitive processes to predict syntactic entities, studies on CE emphasize the memory-related constraints of differential processing difficulty for complex sentences [3,34,35,36,37,38,39].

Processing RC or CE is determined not only by syntactic structures but also by semantic and pragmatic constraints, resulting in the heterogeneous processing difficulty of complex RC and CE sentences among different languages [3,33,39,40]. For example, an experience-based index such as surprisal is an outcome of processing syntax, semantics and pragmatics combined. Thus, they cannot dissociate the syntactic effects of sentence comprehension from semantics or pragmatics. Moreover, many languages with varying word orders (e.g., Subject-Object-Verb or Subject-Verb-Object) also differ in their way of resolving ambiguities with divergent semantic, pragmatic, and syntactic rules [41]. For instance—unlike English—Korean, Japanese, and Chinese have prenominal RC, do not use a relative pronoun, and process gaps in a different manner. CE in English requires more processing load than canonical structures [42]. CE in Korean and Japanese are necessary when the RC modifies a complex noun phrase [36,43,44]. Furthermore, considerable findings in previous studies are limited in their generalizability across languages, because experiments were conducted in an unnatural environment such as using artificial grammar [32]. Taken together, previous studies have some limitations with respect to investigating syntactic processing in RC and CE separately from semantics and pragmatics across different natural languages.

In the present study, we used Korean gapless RC to investigate how RC and CE differ in processing costs. A gapless RC modifies the noun of the main clause without a syntactic movement of a relative pronoun since there is no gap in the subordinate clause [45]. Using gapless RC, we were easily able to eliminate the controversial issues of where the gap resides and how the syntactic movement of a relative pronoun occurs in RC. Moreover, since Korean RC do not use relative pronouns, we were able to construct a complex sentence with two sets of a subject, an object, and a verb, resulting in a complex six-word sentence across experimental conditions. Additionally, Korean is a head-final language using case markers to indicate the grammatical role of a word such that Korean can be easily scrambled as long as the verb is positioned at the end of the sentence [46]. This flexibility of Korean has the advantage of creating center embedded complex sentences with less ambiguity. Notably, Korean RC is center embedded when it acts as a premodifier in a complex noun phrase [44,47], which allows us to create complex six-word sentences that have both an RC and CE. An additional advantage of the flexibility of Korean is the ability to create sentences devoid of commas because separating clauses does not heavily depend on commas. Using commas can be problematic based on the fact that participants might recognize a sentence structure by just detecting commas instead of actively being involved in sentence comprehension. Accordingly, using the gapless RC structure combined with the flexibility of Korean, we were able to make four different experimental conditions with each sentence varying its RC and CE while being of identical length (6 words) and having identical word categories (two subjects, two objects, and two verbs). 

It has been shown that the flexibility of word order in Korean necessitates a semantically and/or pragmatically affordable relationship between an RC and a head noun phrase, and this renders the structure context-sensitive [46,48,49]. The use of linguistic as well as non-linguistic and real-world knowledge has been known to influence on interpreting relative clauses [46]. Therefore, to minimize unwanted influences of semantics and pragmatics in the processing of RC and CE and to reduce lexical surprisal effects [50], we used a newly adopted self-paced pseudoword reading task by replacing every content word with a pseudoword, leaving only legitimate function words (i.e., case markers) in each sentence. In the present study, the first experiment using the conventional self-paced reading task will, therefore, reveal processing differences generated by different syntactic structures under the influence of the semantic and pragmatic contexts created by real words and their combinations. More interestingly, the second experiment using the new self-paced pseudoword reading task will demonstrate a structure-specific effect independent of semantic and pragmatic influence. To the best of our knowledge, we are the first to examine the reading time pattern of processing complex sentences comprised of only pseudowords and function words. 

In the present study, we were able to observe how the RC and CE interplay, producing four distinctive processing patterns from an identical set of constituents across conditions. The effect of RC involved its integration with the head noun, resulting in increased reading time for the head noun. The effect of CE elicited an increase in reading time for the object of the main clause because the parser should retrieve the structure of the main clause. Interestingly, the interaction of RC and CE was significant, and the coexistence of the two operations in a structure did not increase difficulty accordingly. Reading times for the sentences with both RC and CE were shorter than those of the sentences with RC alone. We further investigated the frequency of the two structures and found that the condition with both RC and CE was almost seven-fold more frequent than its counterpart suggesting validity of experience-based account even for purely syntactic processing.

## 2. Experiment 1

### 2.1. Materials and Methods

#### 2.1.1. Participants

Thirty-five native speakers of Korean (19 females, mean age = 22.51; SD = 3.59) participated in the experiment. All participants had a normal or corrected-to-normal vision. They completed the reading span test [51] with Korean sentences [52], and only those who scored more than three could participate in the experiment, resulting in the final 30 participants (15 females, mean age = 22.25, SD = 3.5). All participants were right-handed as assessed by the Edinburgh Inventory [53]. The experiment was conducted in accordance with the recommendations of the Daegu Gyeongbuk Institute of Science and Technology (DGIST) ethics committee, and the DGIST ethics committee approved this study. Every participant was informed about the possibility of withdrawing from the experiment without any disadvantage, signed a written informed consent form accordingly, and received cash compensation of 30,000 KRW. 

#### 2.1.2. Experimental Design

**Figure 1:**
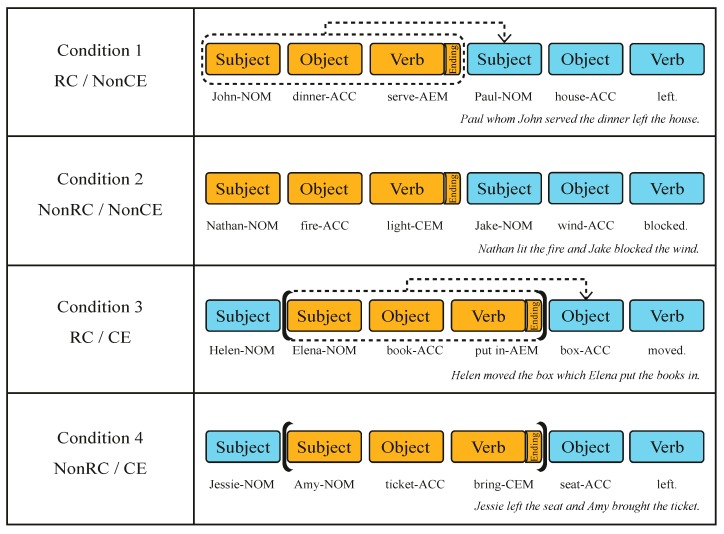
Four different conditions of Experiment 1. Blue and orange shades represent the main clause and the subordinate clause, respectively. Ending markers (an AEM or CEM) are shown as “Ending” only after the first verb for easy explanation here. Case markers attached to each subject, object, and verb were shown throughout the actual experiment. Dotted arrows indicate the formation of an RC between clauses. Brackets surrounding subordinate clauses indicate CE. Condition 1 has an RC in two clauses, with the first clause modifying the subject of the second clause (Paul-NOM). Condition 2 has two clauses conjoined by a CEM without an RC or CE. Condition 3 has both an RC and CE. In this case, the subordinate clause (a structure shaded in orange) is merged with the following main object (box-ACC). Condition 4 has the only CE with a CEM. Here we have provided English names to aid understanding of the subjects; Korean names were used in the actual experiment. Abbreviations: RC (relative clause), CE (center embedding), AEM (adnominalizing ending marker), CEM (conjunctive ending marker), NOM (nominative case marker), ACC (accusative case marker).

Using Korean sentences, we crossed two factors, the relative clause (RC versus NonRC) and center embedding (CE versus NonCE), resulting in four experimental conditions of RC/NonCE, NonRC/NonCE, RC/CE, and NonRC/CE (Figure 1). We used the same combination of word constituents, that is, two subjects, two objects, and two verbs within each sentence across every condition, avoiding any different working memory load caused by deploying a different number of words. The type of ending marker attached to the first verb defined how the two clauses were conjoined or merged. An adnominalizing-ending marker (AEM) was used to create the syntactic dependency in sentences with an RC [54], and a conjunctive ending marker (CEM) was used to generate sentences with a NonRC [55]. Syntactic dependency indicates relations between words having asymmetrical relations; one word is subordinated (dependent) on the other (i.e., head), and each one imposes its selection restrictions [56].

Relative Clause (RC). The RC was induced by using an ending marker that defines a restrictive syntactic dependency between two clauses. Each sentence was composed of two clauses using either an AEM or a CEM. An AEM was attached to the verb of the subordinate clause that modified the following subject or the object of the main clause, making this subordinate clause a relative clause for RC conditions. For conditions without the RC (NonRC), two clauses were joined by a CEM, and thus the preceding clause did not modify the following clause. 

Center embedding (CE). Inserting a subordinate clause between the main subject and the main object created the sentences with the CE. A formal way to address CE in Korean is to insert commas at the beginning and the end of the embedded clause. However, this poses a problem in that participants could recognize embedded structures not by comprehending the sentence but by simply looking at commas. Therefore, instead of inserting commas, we only used case markers and were still able to make grammatical sentences in Korean. To ensure that all the stimuli read well without any problems, we measured the acceptability of every experimental sentence. A seven-point scale was used [57] with a score of seven being “totally acceptable” and a score of one being “totally unacceptable.” Data were collected online from 41 undergraduate students via SurveyMonkey (SurveyMonkey Inc., San Mateo, California, USA, www.surveymonkey.com). The mean acceptability rating of every stimulus was 5.16 (*n* = 160, SD = 0.623). More specifically, the mean acceptability rating of the sentences with CE that had no commas was 5.50 (*n* = 80, SD = 0. 634). This indicates that the acceptability of CE conditions without commas was comparable to other conditions without triggering unexpected effects while reading the sentences. 

#### 2.1.3. Materials

A total of 160 Korean sentences were used as experimental stimuli for the experiment. We used conventional Korean names, instead of occupational nouns (e.g., police officer, teacher, or lawyer), as subjects in the sentences to avoid an unintended influence of word frequency or unwanted build-up of semantic relationships between words. All the names were derived from a sociolinguistic study on Korean names [58]. Every name had two syllables and appeared only once throughout the experiment. Moreover, names were counterbalanced for gender and final syllable type, which determined their following case markers (i.e., either an -*ika* or a *-ka*). These nominative case markers were also counterbalanced by varying the order and number of their appearance in stimuli (e.g., -*ika* for the first subject and -*ka* for the second subject) to neutralize the morphological and phonological differences that might cause interference [59]. All the words used for objects or verbs were acquired from the Sejong Modern Korean Balanced Corpus [60], which consists of 10 million different words. We only used words that appeared more than 100 times in the corpus. Forty sentences were created for each condition, resulting in a total number of 160 experimental sentences. 

Additionally, we created 160 filler sentences with an equal number of words (six words), but with a different combination of constituents (sentences with one subject or one verb, etc.). This was possible due to the pro-drop nature of Korean, which allows the omission of words (a subject, an object, or a verb) when readers can deduce omitted words from grammar or the context of the sentence [61]. Adjectives and adverbs substituted an excluded subject, a verb, or an object. Fillers composed of words from the same corpus with identical counterbalancing criteria for word frequency, names used as subjects, use of case markers, and word length as in the experimental sentences. Moreover, we added 34 filler sentences, which were longer than the experimental sentences (7 to 10 words). The examples of sentences in Figure 1 are provided in Korean using Yale Romanization [62] below (Table 1).

#### 2.1.4. Procedure

Participants performed the self-paced reading task via a non-cumulative word-by-word moving-window paradigm on a desktop PC. We used PsychoPy to present stimuli [63]. Participants were presented with a dashed line on a screen, and by pressing the space bar on a keyboard, they revealed the first word of the sentence and consecutively pressed it to reveal the next word, simultaneously masking the previously shown word. Thus, participants could read the whole sentence word-by-word, and the reading time of each word could be measured by intervals between the key presses. Participants were told to read sentences as naturally as possible. After the last word of the sentence was revealed, and the space bar was pressed, a comprehension question composed of a shortened sentence was presented for two seconds. Participants were asked to judge whether the shortened sentence expressed the same content with the previously presented sentence. This way, we were able to ensure that participants actively engaged in understanding the stimulus sentence. The shortened sentence was constructed by selecting a subject, an object, and a verb from the previous stimulus. Incorrect sentences were generated either by switching the position of words or by replacing one word with a new word that was not present in the stimulus. The whole experiment took approximately 40 min with a 5-min break halfway through.

#### 2.1.5. Data Analysis

Only correct answers were used for the analysis. Reading times below 100 ms or above 3500 ms that were known to be uninformative [64] were discarded. Reading times with more than three standard deviations were also discarded. This resulted in a loss of 1.46% of the data (384 from 26,262 observations). For every graph depicting means and 95% confidence intervals, we followed methods developed by Loftus and Masson, simplified by Cousineau, and again corrected by Morey [65,66,67]. The trimmed data underwent a separate linear mixed-effect analysis for each position of the word or each constituent of the clause, using the lme4 package [68,69] in R software (version 3.4.3). Concerning the linear mixed-effect analysis in reading time data, there is controversy over the use of either a maximal model or the simplest model for fitting the data [70,71,72,73]. Following the recommendation by Matuschek and his colleagues, we included random slopes and chose the best model fit with the lowest Akaike information criterion [73]. Accordingly, by-subject intercepts and slopes were fitted for each word position as random effects. Models without by-item random effect were expected to show better fits since every stimulus was classified under only one condition [70]. To minimize the autocorrelation caused by the series of words [13,74,75,76], preceding word positions were fitted as a fixed effect for the target word position (e.g., word 1 and word 2 for the target position word 3). To control various word lengths, a length value (i.e., the number of characters in a given word) for every word was separately coded and fitted as a fixed effect. We compared four conditions using two-by-two linear mixed-effect analyses for every word position. 

For accuracy of the comprehension questions, we used a classical repeated-measure two-way ANOVA and a linear mixed-effect analysis [70]. The same lme4 package from R software was used to analyze the data from comprehension questions [69]. The random structure with a by-subject intercept and a slope was adopted. 

The reaction time (RT) of comprehension questions underwent the repeated-measure two-way ANOVA and linear mixed-effect analysis. The random structure, including the by-subject intercept and slope for interaction term, did not fail to converge. The lme4 package recently removed the post hoc Markov-chain Monte Carlo method for calculating a *p*-value due to its unreliability [69]. Therefore, we used the lmerTest package to calculate *p*-values [77]. 

### 2.2. Results

#### 2.2.1. Comprehension Questions

The mean and standard error of both the accuracy and RTs for the comprehension questions are shown in Table 2. There were no significant main effects or the interaction by the RC and CE on RT and accuracy with the repeated-measure two-way ANOVA. The linear mixed-effect analysis on RT and accuracy did not show significant effects either.

#### 2.2.2. Reading Times

**Figure 2:**
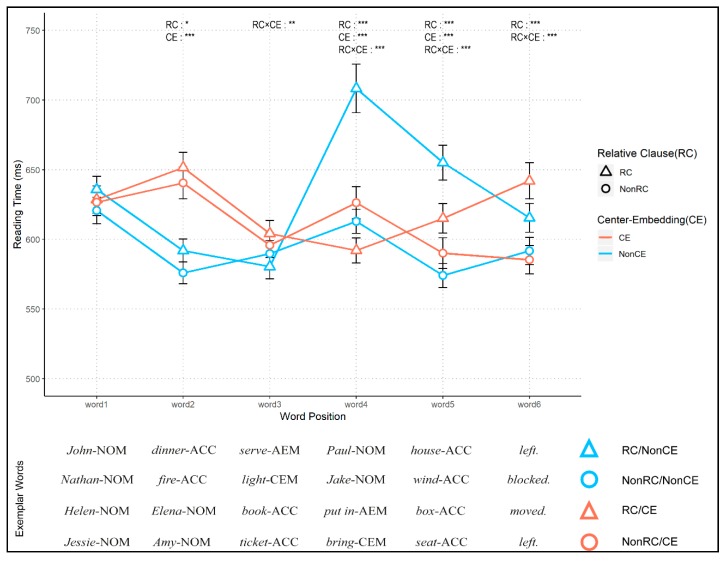
Reading-time data from the self-paced reading task in Experiment 1. The mean reading times with standard deviations are depicted for all the conditions. The x-axis indicates the position of words presented, and the y-axis denotes the reading times in milliseconds (ms). The triangular data points depict the RC condition, while the circular data points depict the non-RC (NonRC) condition. Blue and orange lines indicate non-CE (NonCE) and CE conditions, respectively. The significant effects from two-by-two linear mixed-effect analyses are shown at the top of each word position (For details, see Table 3). Exemplar words labeled with four conditions (i.e., RC/NonCE, NonRC/NonCE, RC/CE, NonRC/CE) are presented at the bottom. Abbreviations: RC (the main effect of a relative clause), CE (the main effect of center embedding), RC × CE (an interaction between the relative clause and center embedding), AEM (adnominalizing ending marker), CEM (conjunctive ending marker), NOM (nominative case marker), ACC (accusative case marker). (* = *p* < 0.05, ** = *p* < 0.01, *** = *p* < 0.001).

The overall reading time of the four conditions is shown in Figure 2. As mentioned in the data analysis, confident intervals for every graph were calculated by the method appropriate for repeated-measure designs.

Table 3 shows parameter estimates, *t*-values, and *p*-values of the three key fixed effects (main effects of RC and CE, and their interaction). There was no significant difference in the initial word position (word 1). However, for word 2, the main effect of CE was significant (*t* = 12.325, *p* < 0.001) and the main effect of RC was marginally significant (*t* = −2.026, *p* = 0.043). There was no significant interaction (RC × CE) for word 2. For word 3, a significant interaction (*t* = −2.680, *p* = 0.007) was observed, whereas no main effects were found for RC and CE. Notably, highly significant interactions were observed for word 4 and word 5 (word 4, *t* = 11.065, *p* < 0.001; word 5, *t* = 4.585, *p* < 0.001). Likewise, the main effects of words 4 and 5 were significant. Lastly, the main effect of RC and the interaction between RC and CE were significant for word 6 (RC, *t* = −4.436, *p* < 0.001; RC × CE, *t* = −5.009, *p* < 0.001).

### 2.3. Discussion

In Experiment 1, the four conditions showed different reading time patterns depending on their sentence structures. The first three words (words 1, 2, and 3) showed similar patterns in reading time between RC and NonRC in both CE and NonCE conditions. Word 1 followed either a subject or an object depending on the presence of CE, which resulted in different reading times for word 2 between CE and NonCE. For reading time patterns of CE conditions (RC/CE and NonRC/CE; orange lines in Figure 2), word 2 had the longest reading time compared with the rest (words 3, 4, 5, and 6), because probabilistic uncertainty was at its peak for word 2 and gradually decreased as other word categories were revealed towards the end of each sentence. Based on the similarity-based interference proposal [38], the increased reading time in word 2 could be interpreted as interference by a consecutive presentation of the same noun phrases, which was observed in an eye-tracking study of Korean [59]. For word 3, the reader used different syntactic information to process each condition. While the first objects (e.g., book-ACC for RC/CE and ticket-ACC for NonRC/CE in Figure 2) were introduced in CE conditions in word 3, initial verbs with different markers (e.g., serve-AEM for RC/NonCE and light-CEM for NonRC/NonCE in Figure 2) were introduced in NonCE conditions. This led to a small but significant interaction effect in word 3.

For word 4, the two main effects of RC and CE with their interactions were all significant. A noticeable increase in reading time for word 4 in the RC/NonCE condition may be due to the interaction of surprisal from a new subject and the establishment of dependency (RC). When comparing the RC/NonCE with NonRC/NonCE, both sentences had new subjects for word 4 and thus showed increased reading times compared to those at word 3. However, the degree of increase in reading times was much higher for RC/NonCE than for NonRC/NonCE. This observation points out that readers, with the help of AEM, make relativization between the main clause and the subordinate clause in RC/NonCE [54], incorporating the two clauses into a more complex sentence compared to NonRC/NonCE. Notably, the difference between the two conditions in word 4 cannot be explained by the type of information processed, as they have identical word categories (NOM), or by the amount of information stored or manipulated, as they have an equal number of words.

Interestingly, the RC effect for word 4 worked differently in CE conditions (the RC/CE and NonRC/CE), leading to a significant interaction between RC and CE. The longer reading time for word 4 in the NonRC/CE condition means that the embedded clause without modifying any words of the main clause was more surprising to the readers than the embedded clause modifying the object of the main clause (RC/CE). Embedding a clause without making a relative clause is unlikely to happen. Critically, a syntactic dependency between two clauses, which is indicated by a case marker, recruits additional cognitive resources independent of the order, amount, or type of words stored and manipulated during sentence processing. This increased reading time for different case markers (AEM vs. CEM) suggests that the parser is already distinguishing the RC and NonRC structure. In the current experiment, however, this effect is not clear between the two NonCE conditions for word 3. Therefore, the current result only supports the claim that disambiguation takes place at the head noun [17,46,61,78]. It is noteworthy that this observation might be a particular case for a right-branching and head-final language that adopts a case marker system. For left-branching languages such as English, it is relatively difficult to dissociate the effect of word order and relative pronouns from cues that indicate a relative clause. Korean syntax, on the other hand, enables manipulation of such cues without changing the word order or sentence length. Notably, it has been known that Korean has a relatively free word order [79], which is facilitated by various case markers. The number of case markers in Korean is known as eight, including nominative, accusative, genitive, dative, locative, instrumental, comitative, and vocative. These case markers enable Korean to be flexible in the ordering of subject and object arguments as in Japanese, Turkish, or German [80]. 

Furthermore, the processing difficulty addressed by hierarchy formation can be observed in words 5 and 6. Provided that readers expected an object or a verb to appear for words 5 and 6, the surprisal of the four conditions would amount to a similar level, and thus reading time of the four conditions would gradually converge. Nevertheless, RC conditions (RC/NonCE and RC/CE) showed longer reading times compared to NonRC conditions (NonRC/NonCE and NonRC/CE), having the significant main effect of RC. Moreover, unlike the NonCE conditions, where both the reading times of RC and NonRC conditions became similar at the end of the sentence (word 6), the difference in reading times between RC/CE and NonRC/CE increased further, differentiating the two conditions towards the ends of the sentences. This might be due to the differential complexity of the sentence structures between the RC and NonRC conditions. Although the uncertainty was further resolved for the last two words, processing hierarchical structures required additional time even at the ends of the sentences in the RC conditions.

Despite controlling the four conditions as equally as possible only to have a syntactic difference, the semantic influence during sentence comprehension cannot be completely ruled out. For example, the relationship between an object and a verb in a sentence might have triggered a close semantic relation as a confounding effect in the present study. Moreover, although the RC manipulation was derived by a phonologically and morphologically identical suffix, semantic and pragmatic constraints for RC/NonCE and RC/CE were not identical between the two conditions. Our sentences for the RC/CE constitute center embedded and gapless RC without the syntactic requirement of an additional noun. However, sentences for the RC/NonCE did require an adverb, that is, a ‘vital adverb’ in Korean. Korean vital adverbs are suffixed not by an accusative marker but by an adverbial marker, which is why they are termed as ‘adverbs.’ Interestingly, this is semantically comparable to an indirect object in English such that subordinate verbs in RC/NonCE function as semantically dative verbs. In other words, RC in Korean cannot be explained by a traditional view of RC in English [81,82]. Therefore, considering all the arguments mentioned above, we conducted a pseudoword version of the self-paced reading task in the second experiment to further control for unexpected effects of semantics. 

## 3. Experiment 2

### 3.1. Materials and Methods

#### 3.1.1. Participants

Thirty-two native speakers of Korean (16 females, mean age = 20.19; SD = 1.89) participated in the experiment. All had a normal or corrected-to-normal vision. Criteria for excluding participants were identical to that of Experiment 1. However, those who performed with an accuracy of less than 70% in the pseudoword self-paced reading task were excluded from the analysis, resulting in the final 20 participants (9 females, mean age = 19.95, SD = 1.59).

#### 3.1.2. Materials

**Figure 3:**
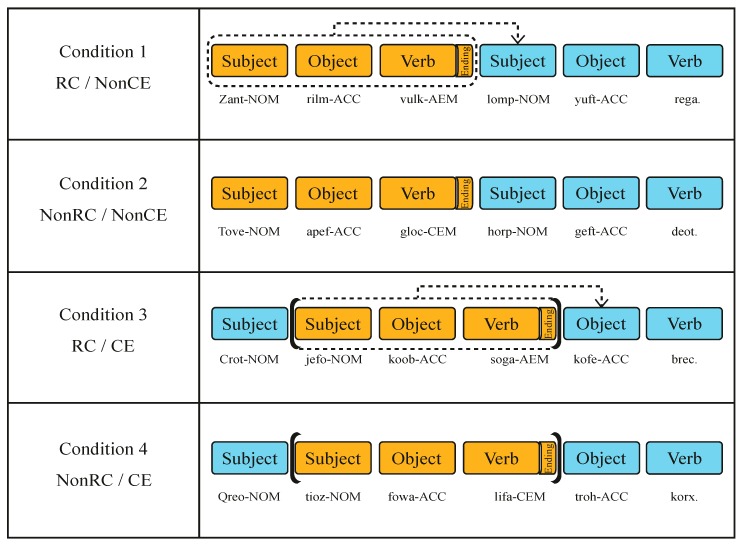
The pseudoword version of stimuli in Experiment 2. Blue and orange shades represent the main clause and the subordinate clause, respectively. Ending markers (an AEM or a CEM) are shown as “Ending” only after the first verb for easy explanation here. Case markers attached to each subject, object, and verb were shown throughout the actual experiment. Dotted arrows indicate the RC. Brackets surrounding the subordinate clause indicate the CE. Construction of every condition was identical to that of Figure 2. Pseudowords did not provide participants with cues for a constituent—whether the word was a noun or a verb. Therefore, they could only infer the syntactic role of the pseudowords by case markers or ending markers attached to the pseudowords. Here we have provided English pseudowords with four letters to aid understanding of the stimuli. In the actual experiment, we used Korean two-syllable pseudowords. Abbreviations: RC (relative clause), CE (center embedding), AEM (adnominalizing ending marker), CEM (conjunctive ending marker), NOM (nominative case marker), ACC (accusative case marker).

We designed sentences with pseudowords in a fashion that content words of sentences in Experiment 1 were replaced with pseudowords. The number of stimuli was identical to that of Experiment 1. We created pseudowords by decomposing every word from the list in The Korean Lexicon Project [83] into syllables, sampling two syllables randomly, and concatenating them into a two-syllable pseudoword. By using the syllables already adopted in real words from the Korean lexicon, we successfully created pseudowords with syllables that obey the orthographic and phonologic rules of Korean. Besides, we screened out some combinations of randomly sampled syllables, which happened to be real words. We created 5000 pseudowords, and three Korean native speakers scrutinized the list to exclude further pseudowords that resembled real words or neologisms. Finally, 1380 pseudowords were selected. We created 40 pseudoword sentences for each of the four conditions as in Experiment 1 and 30 pseudoword sentences for the practice session. Additionally, a dummy condition that consisted of pseudowords was included to mimic the role of filler sentences in Experiment 1. Every sentence had six pseudowords included within a sentence. The manipulation of ending markers and structures were adopted in the same way as Experiment 1 (Figure 3).

#### 3.1.3. Procedure

The procedure was mainly identical to that of Experiment 1. We chose words cautiously when instructing participants about the task and avoided informing them directly that function words (case markers and ending markers) were intact. This prevented participants from intentionally focusing on function words to grasp the structure of the sentence early in the trials. Participants were asked to read pseudoword sentences as naturally as possible. Thirty practice trials were given to participants before the main task. Comprehension questions were created and asked in the same manner as Experiment 1, with one exception: participants had to decide within three seconds (two seconds in Experiment 1).

#### 3.1.4. Data Analysis

The data analysis in Experiment 2 was mostly identical to that of Experiment 1, with one exception: Given that we used two-syllable pseudowords, we no longer needed to regress out the influence of word length for the analysis. Trimming of outliers resulted in a loss of 1.31% of the data (190 from 14,544 observations). The data from the comprehension question was analyzed in the same way as Experiment 1. The random structure with a by-subject interaction effect failed to converge for RT analysis, and thus we chose the alternative model without the interaction term in the random structure.

### 3.2. Results

#### 3.2.1. Comprehension Questions

The mean and the standard error of both the accuracies and the RTs for the comprehension questions are shown in Table 4. For the accuracy, a classical repeated-measure two-way ANOVA showed no significant effects. However, linear mixed-effect analysis for accuracy showed significant main effects of the RC (*t* = 6.403, *p* < 0.001) and CE (*t* = −6.088, *p* < 0.001). No significant interaction effect was found (RC × CE, *t* = 0.351, *p* = 0.729). In the RT data, a two-way ANOVA revealed only the main effect of CE (*F* = 9.509, *p* = 0.00206). The linear mixed-effect analysis showed a significant main effect of CE (*t* = 6.571, *p* < 0.001) and RC (*t* = −5.727, *p* < 0.001). The interaction was not significant. Interestingly, RC/CE condition had the lowest accuracy compared to other conditions, which may be related to additional costs for comprehending RC/CE sentence. 

#### 3.2.2. Reading times

**Figure 4:**
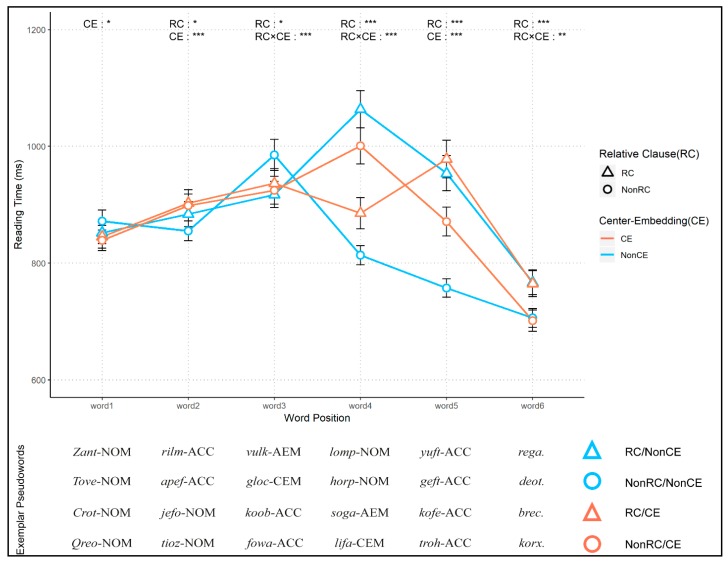
Reading time data from the self-paced pseudoword reading task in Experiment 2. The mean reading times with standard deviations are depicted for all the conditions from Experiment 2. The x-axis indicates the position of pseudowords presented, and the y-axis denotes the reading times in milliseconds (ms). The triangular data points depict the RC condition, while the circular data points depict the non-RC (NonRC) condition. Blue and orange lines indicate non-CE (NonCE) and CE conditions, respectively. The significant effects from two-by-two linear mixed-effect analyses were depicted at the top of each word position (for details see Table 5). Exemplar pseudowords labeled with the four conditions (i.e., RC/NonCE, NonRC/NonCE, RC/CE, NonRC/CE) are presented at the bottom. Abbreviations: RC (the main effect of relative clauses), CE (the main effect of center embedding), RC × CE (an interaction between the relative clause and center embedding), AEM (adnominalizing ending marker), CEM (conjunctive ending marker), NOM (nominative case marker), ACC (accusative case marker). (* = *p* < 0.05, ** = *p* < 0.01, *** = *p* < 0.001).

The overall reading time data from Experiment 2 are shown in Figure 4. 

Table 5 shows parameter estimates, *t*-values, and *p*-values for key fixed effects. For word 1, there was a marginally significant effect of CE (*t* = −2.112, *p* = 0.035), but no other effects (RC, RC × CE) were significant. The main effect of CE increased in word 2 (*t* = 4.626, *p* < 0.001), while the effect of RC was still marginally significant (*t* = −2.329, *p* = 0.020). The interaction between CE and RC was not significant for word 2. However, the main effect of RC and the interaction between RC and CE were significant for word 3 (RC, *t* = 2.350, *p* = 0.01886; RC × CE, *t* = −3.729, *p* < 0.001). For word 4, the same effects were found more strongly (RC, *t* = −5.430, *p* < 0.001; RC × CE, *t* = 15.050, *p* < 0.001). However, for word 5, the interaction effect disappeared (RC × CE, *t* = −0.004, *p* = 0.997), whereas the two main effects survived (RC, *t* = −11.615, *p* < 0.001; CE, *t* = 5.608, *p* < 0.001). Lastly, for word 6, the main effect of RC (*t* = −4.912, *p* < 0.001) and the interaction effect (*t* = −2.857, *p* = 0.004) were found.

### 3.3. Discussion

Implementing pseudowords in a self-paced reading paradigm increased the overall processing difficulty, which resulted in a one-and-a-half-fold increase in the reading times and some significant main effects of experimental manipulations (the RC and CE) for the accuracy and RT of comprehension questions. These observations imply that semantics in the stimulus sentences must have played a critical role in supporting sentence comprehension in Experiment 1. However, the pattern of the reading time acquired from Experiment 2 mostly corroborates the results of Experiment 1. For instance, a significant main effect of CE for word 2 reaffirms that consecutive appearance of the second subject increases the reading time in this word position. Additionally, for two CE conditions (RC/CE and NonRC/CE), the reversed pattern of reading times in words 4 and 5—where the reading time of RC/CE increased, while the reading time of NonRC/CE decreased—was also replicated in Experiment 2. Despite the decrease in reading times across RC/CE and NonRC/CE, this effect of RC even remained until word 6. 

Interestingly, the reading time patterns from Experiment 2 show several discriminative features compared to that of Experiment 1. Overall, reading times tended to decrease across the conditions as participants read toward the end of the sentence. This indicates that readers resolve syntactic ambiguity by the end of the sentence, which is also known as anti-locality observed in many head-final languages, including Korean [12,84]. Furthermore, even though the reversed pattern of reading times in words 4 and 5 in Experiment 1 was replicated here, the overall reading time of this pattern increased compared to the reading time of the NonRC/NonCE condition in Experiment 2. This finding suggests that processing word sequences deployed by CE imposes additional cognitive resources, as the reader must retrieve the main clause while reading words 4 and 5 to complete a long-distance dependency. Moreover, the fact that this effect was more apparent in Experiment 2 than in Experiment 1 indicates that the processing of semantic information in the sentence might support the retrieving process.

Lastly, the differential effect of case markers, which was only observable for the two CE conditions on word 4 in Experiment 1, is also observed for the two NonCE conditions on word 3. Unlike Experiment 1, the subordinate verb (word 3 of the two NonCE conditions) followed by an AEM always showed a shorter reading time compared to the verb that was followed by a CEM, regardless of the presence of CE. Along with this finding, the head nouns (i.e., words 5 and 6 for NonCE and CE conditions respectively) that follow these case markers (i.e., subjects following an AEM) consistently showed longer reading time compared to their counterparts (i.e., subjects following a CEM). Taken together, these findings extend the previous findings on Korean RC, which determine the head noun as the locus of disambiguation [17,46,61,78] and provide additional evidence that the prenominal case marker (word 3 for NonCE conditions and word 4 for CE conditions) also aids the parser’s disambiguation of the structure [31].

## 4. General Discussion

The present study investigated the processing cost of syntactic operations required for RC and CE by fully utilizing the flexibility of Korean syntax. Moreover, we took advantage of the pseudoword self-paced reading task and thus reduced unnecessary influences of semantics or pragmatics on sentence processing, only focusing on syntactic features.

### 4.1. Disambiguation in Korean RC

Previous studies on Korean RC have mainly focused on where the disambiguation of the RC structure is resolved and where the difficulty arises [17,31,44,46,59,61,78]. While these findings have generally centered on the head noun as the site of structural integration, a recent study [31] emphasized the AEM as a clause-type disambiguation point. Our results from Experiment 2 reveal both the effect of AEM and its head noun (Figure 4). The effect of the marker (the longer reading time for CEM compared to AEM) in NonCE conditions is clearly visible for word 3, and the same effect is observed for word 4 in CE conditions. Likewise, the effect of a head noun (the longer reading time for the modified head noun compared to the un-modified head noun) was observed in word 4 for NonCE conditions and word 5 for CE conditions. These results support the claim that the cue (AEM) and its modificand (head noun) both serve as disambiguation points [31]. 

### 4.2. Surprisal and Its Relation to Reading Times in RC

It has long been noted that expectation-based approaches favor RC construction, emphasizing the prediction of syntactic entities. Our study also conforms to this argument, showing that the reading time for the RC/CE condition was less than that of the RC/NonCE condition, specifically from word 4. This unexpected finding may be explained by the surprisal theory, which provides clues on interpreting the ease of processing for sentences in the RC/CE condition. 

**Figure 5:**
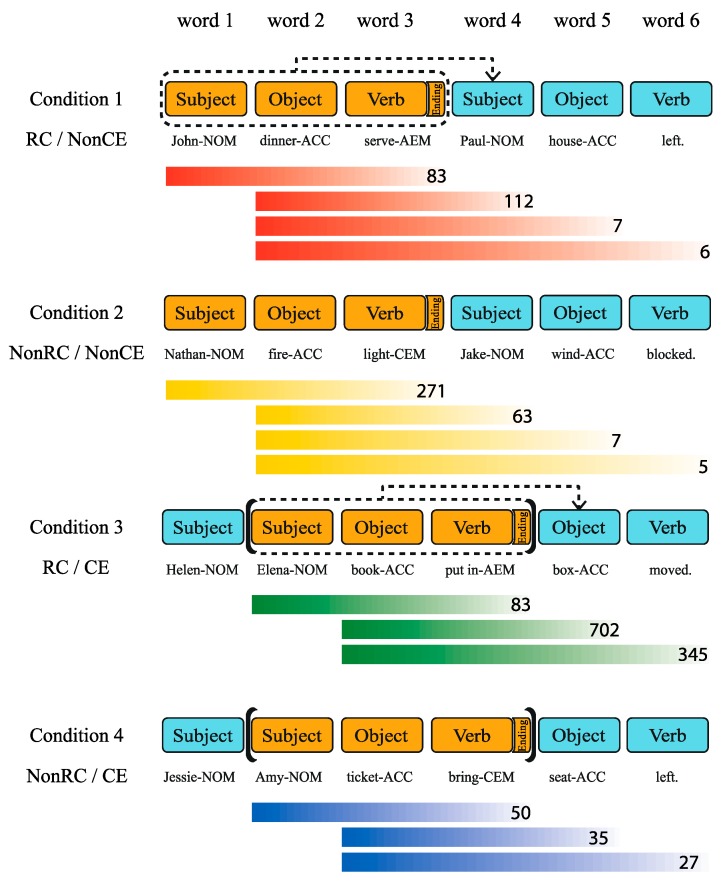
The partial structure frequencies of the stimuli. The number at the right end of the color gradient indicates the frequency of the partial structure (i.e., a set of words covered by the length of the color gradient) occurring in the corpus. For example, “83” in Condition 1 indicates that the structure of (subject + object + verb-AEM) appeared 83 times in the corpus [60]. The first values for Condition 2 and 4 are not identical because morphologically different endings were used. Not every partial structure was used to determine the relative difference in surprisal because only the structures with the same starting position can be compared. The Sejong Semantically Tagged Corpus of the Modern Korean with 797,251 sentences were analyzed by Hanmaru 2.0, a concordance program developed together with the corpus itself, to calculate partial structures of the sentence stimuli. Abbreviations: AEM (adnominalizing ending marker), CEM (conjunctive ending marker), NOM (nominative case marker), ACC (accusative case marker).

Surprisal can be estimated by frequencies of a specific structure in a sentence from a corpus [15]. However, there was no Korean corpus large enough to find the full structure of our sentence stimuli with syntactic tags across the four conditions. Thus, we made the best use of the Korean corpus that is currently available and calculated the frequency of partial structures of our sentence stimuli, presenting a reasonable estimation of surprisal (Figure 5). The subordinate clauses with an identical structure (Subject-NOM + Object-ACC + Verb-AEM) in both RC/NonCE (Condition 1) and RC/CE (Condition 3) had 83 incidents in the corpus. However, it was less usual for the head noun of a subordinate clause to be a subject (112 incidents) in the main clause of RC/NonCE than to be an object (702 incidents) in the main clause of RC/CE. This differential frequency could be the reason for the difference in word 4. The interaction of the RC and CE presenting the object as a head noun was better at meeting the syntactic expectation than RC/NonCE presenting the subject as a head noun. Thus, RC/CE resulted in lower reading times compared to RC/NonCE. This result is in line with previous findings, showing that expectation-based theories are broadly supported in languages such as Chinese [30], Japanese [84], and Korean [17]. 

### 4.3. On-Line Constraints of a Memory Load and Its Relation to Reading Times in CE

The embedding is established by inserting a clause within a superordinate clause [33]. The effect of the embedding is revealed as the increase in the reading time in the main object that needs integration with the retrieved main subject. This process is the hallmark of the embedding, which requires considerable cognitive demands [34,37]. The onset of the main object necessitates parsers not only recalling and integrating the main subject but also dealing with two clauses by incorporating a sequence of words into one complex structure. This effect was observed not only in Experiment 1, but also in Experiment 2 when we minimized the possible interplay of semantics by using pseudowords with the comparison between NonRC/CE and NonRC/NonCE. This could be interpreted as the effect caused by maintaining the information about the main subject while reading the embedded clause to retrieve it later and to integrate it with the main object. The effect of the embedding is demonstrated in words before and after the embedded clause. The information on the main subject should be maintained until it meets its main object/verb and thus imposes additional working memory load on the processing of the embedded clause. In the meantime, the main object after the embedded clause induces higher reading time due to the retrieval of the main subject and the integration afterward. Our results add another behavioral evidence on processing embedded sequences in natural language processing.

### 4.4. Advantages of the Self-Paced Pseudoword Reading Task

Measuring a pattern of reading time with a self-paced reading task has been widely used for the investigation of language comprehension [85]. This method suggests that relatively slow or fast reading times indicate the readers’ processing difficulty or facilitation, respectively [86]. Researchers have used a self-paced reading task to understand the comprehension of a subject-extracted relative clause or object-extracted relative clause [20,87]. In the present study, we conducted a self-paced reading task using pseudowords that have tangible benefits for the study. 

It is inevitable that sentence comprehension is influenced by the interaction of semantics and syntax [88,89]. Linguistic theories concerning RC and CE show a clear difference between Korean and other languages. For example, analyses on gapless RC in English are ambiguous, and thus they have been relatively unacceptable [90]. One explanation on gapless RC in English indicates that the relative pronoun functions as a conjunctive cue rather than a proper relative pronoun [91,92,93,94]. However, gapless RC in Korean, Japanese, and Chinese share an identical morphological cue with noun complement clauses, which has engendered controversy on whether the gapless RC should be classified as an RC or a noun complement clause [48,95,96,97,98,99,100,101,102,103,104]. This controversy often includes the involvement of semantic and pragmatic constraints in a sentence, and thus it cannot be resolved solely by syntax [48]. Therefore, we used pseudowords to minimize the effect of semantics and to look into the structural differences in syntactic comprehension. In the present study, we could reduce semantic information as well as pragmatic information, which enabled us to take advantage of gapless RC with minimal semantic information.

The present study has a potential limitation. We used untransformed RT data, which may yield potential biases associated with skewed RT data. Skewed RT data may influence the estimate of the mean, whereby statistical tests may produce distorted consequences. Therefore, future studies should be carried out to verify the results of the present study with transformed RT data. Alternatively, Generalized Linear Mixed Models [105] may provide a solution to this problem with a better statistical power of the data, satisfying normality assumptions without the need for raw data transformation.

## 5. Conclusions

Our study revealed computational profiles of RC and CE using well-controlled experiments to carefully discard semantic and pragmatic influences on sentence processing, which enabled us to extract purely syntactic elements. Future work using pseudoword reading tasks with different methodologies such as eye-tracking, event-related potential (ERP), and functional magnetic resonance imaging (fMRI) may shed light on finer spatiotemporal information on how RC and CE calculations are processed in the brain. 

## Figures and Tables

**Table 1:** Examples of sentences in Figure 1.

RC/NonCE:	John이	저녁을	사준	Paul이	집을	나섰다.
	John-*i*	cenyek-*ul*	sacwu-n	Paul-*i*	cip-*ul*	na-sess-ta.
NonRC/NonCE:	Nathan이	불을	켰고	Jake가	바람을	막았다.
	Nathan-*i*	pwul-*ul*	Khyess-*ko*	Jake-*ka*	Palam-*ul*	mak-ass-ta.
RC/CE:	Helen이	Elena가	책을	넣은	상자를	옮겼다.
	Helen-*i*	Elena-*ka*	chayk-*ul*	neh-*un*	sangca-*lul*	olm-kyess-ta.
NonRC/CE:	Jessie가	Amy가	표를	가져오자	자리를	떴다.
	Jessie-*ka*	Amy-*ka*	phyo-*lul*	kacyeo-*ca*	cali-*lul*	ttenass-ta.

**Table 2:** The mean RT and mean accuracy for comprehension questions from Experiment 1.

Conditions	RC/NonCE	NonRC/NonCE	RC/CE	NonRC/CE
RT (ms)	1178.008 (12.259)	1175.367 (11.319)	1200.645 (11.814)	1175.217 (11.265)
Accuracy (%)	91 (0.01)	91 (0.01)	92 (0.01)	91 (0.01)

The numbers represent mean values and standard errors (in parenthesis) of the four conditions.

**Table 3:** The parameter estimates, *t*-values, and approximate *p*-values of the effects from Experiment 1.

Words	Effects	Estimate	*t*-Value	*p*-Value
Word 1	RC	−1.984	−0.982	0.326
	CE	0.100	0.050	0.960
	RC × CE	2.566	1.270	0.204
Word 2	RC	−4.156	−2.026	0.043*
	CE	28.575	12.325	< 2 × 10^−16^***
	RC × CE	−1.005	−0.490	0.624
Word 3	RC	3.066	1.582	0.114
	CE	−0.294	−0.145	0.885
	RC × CE	−5.267	−2.680	0.007**
Word 4	RC	−14.314	−5.280	1.36 × 10^−7^***
	CE	−27.023	−8.491	< 2 × 10^−16^***
	RC × CE	30.140	11.065	< 2 × 10^−16^***
Word 5	RC	−22.834	−9.729	< 2 × 10^−16^***
	CE	−10.675	−4.379	1.22 × 10^−5^***
	RC × CE	10.917	4.585	4.68 × 10^−6^***
Word 6	RC	−10.482	−4.436	9.39 × 10^−6^***
	CE	4.451	1.862	0.063
	RC × CE	−11.769	−5.009	5.71 × 10^−7^***

The estimate column describes the fixed effect parameter estimates of the RC, CE, and RC × CE. Type III sums of squares were used for the analyses. (* = *p* < 0.05, ** = *p* < 0.01, *** = *p* < 0.001).

**Table 4:** The mean RT and mean accuracy for comprehension questions from Experiment 2.

Conditions	RC/NonCE	NonRC/NonCE	RC/CE	NonRC/CE
RT (ms)	1644.465 (23.489)	1515.466 (18.451)	1760.705 (23.807)	1673.426 (21.209)
Accuracy (%)	75.875 (0.015)	88.125 (0.011)	62.75 (0.017)	76.25 (0.015)

The numbers represent mean values and standard errors (in parenthesis) of the four conditions.

**Table 5:** The parameter estimates, *t*-values, and approximate *p*-values of the effects from Experiment 2.

Words	Effects	Estimate	*t*-Value	*p*-Value
Word 1	RC	0.614	0.144	0.886
	CE	−9.005	−2.112	0.035*
	RC × CE	−7.045	−1.653	0.099
Word 2	RC	−10.124	−2.329	0.020*
	CE	20.123	4.626	3.93 × 10^−6^***
	RC × CE	6.336	1.458	0.145
Word 3	RC	13.012	2.350	0.019*
	CE	−10.364	−1.865	0.062
	RC × CE	−20.626	−3.729	0.0002***
Word 4	RC	−32.893	−5.430	6.25 × 10^−8^***
	CE	1.286	0.212	0.832
	RC × CE	91.230	15.050	< 2 × 10^−16^***
Word 5	RC	−69.145	−11.615	< 2 × 10^−16^***
	CE	33.287	5.608	2.3 × 10^−8^***
	RC × CE	−0.024	−0.004	0.997
Word 6	RC	−21.075	−4.912	9.67 × 10^−7^***
	CE	−6.558	−1.566	0.117
	RC × CE	−12.431	−2.857	0.004**

The estimate column describes the fixed effect parameter estimates of the RC, CE, and RC × CE. Type III sums of squares were used for the analyses. (* = *p* < 0.05, ** = *p* < 0.01, *** = *p* < 0.001).
